# Effects of coenzyme Q10 supplementation on antioxidant capacity and inflammation in hepatocellular carcinoma patients after surgery: a randomized, placebo-controlled trial

**DOI:** 10.1186/s12937-016-0205-6

**Published:** 2016-10-06

**Authors:** Hsiao-Tien Liu, Yi-Chia Huang, Shao-Bin Cheng, Yin-Tzu Huang, Ping-Ting Lin

**Affiliations:** 1 Graduate Program in Nutrition, Chung Shan Medical University, Taichung, 40201 Taiwan; 2 Division of General Surgery, Department of Surgery, Taichung Veterans General Hospital, Taichung, 40705 Taiwan; 3 Department of Nutrition, Chung Shan Medical University, Taichung, 40201 Taiwan; 4 Department of Nutrition, Chung Shan Medical University Hospital, Taichung, 40201 Taiwan

**Keywords:** Coenzyme Q10 supplementation, Oxidative stress, Antioxidation, Inflammation, Hepatocellular carcinoma, Surgery

## Abstract

**Background:**

It has been reported that higher levels of oxidative stress and inflammation play a key role in the progression of hepatocellular carcinoma (HCC) after surgery. Coenzyme Q10 is an endogenous lipid-soluble antioxidant. To date, no intervention study has investigated coenzyme Q10 supplementation in HCC patients after surgery. The purpose of this study was to investigate oxidative stress, antioxidant enzymes activity, and inflammation levels in HCC patients after surgery following administration of coenzyme Q10 (300 mg/day).

**Methods:**

This study was designed as a single-blinded, randomized, parallel, placebo-controlled study. Patients who were diagnosed with primary HCC (*n* = 41) and were randomly assign to a placebo (*n* = 20) or coenzyme Q10 (300 mg/day, *n* = 21) group after surgery. The intervention lasted for 12 weeks. Plasma coenzyme Q10, vitamin E, oxidative stress antioxidant enzymes activity and inflammatory markers levels were measured.

**Results:**

The oxidative stress (*p* = 0.04) and inflammatory markers (hs-CRP and IL-6, *p* < 0.01) levels were significantly decreased, and the antioxidant enzymes activity was significantly increased (*p* < 0.01) after 12 weeks of coenzyme Q10 supplementation. In addition, the coenzyme Q10 level was significantly negatively correlated with the oxidative stress (*p* = 0.01), and positively correlated with antioxidant enzymes activity (SOD, *p* = 0.01; CAT, *p* < 0.05; GPx, *p* = 0.04) and vitamin E level (*p* = 0.01) after supplementation.

**Conclusion:**

In conclusion, we demonstrated that a dose of 300 mg/d of coenzyme Q10 supplementation significantly increased the antioxidant capacity and reduced the oxidative stress and inflammation levels in HCC patients after surgery.

**Trial registration:**

Clinical Trials.gov Identifier: NCT01964001

## Background

The most recent World Health Organization (WHO, 2014) report indicated that liver cancer is the second leading cause of cancer deaths worldwide, and 0.8 million people (9.1 % of the global population) died from liver cancer [1]. In Taiwan, liver cancer is also the second leading cause of cancer deaths among men and women (Ministry of Health and Welfare, 2014). Hepatocellular carcinoma (HCC) accounts for most liver cancers [2]. The major risk factors for HCC include hepatitis B or C virus, alcoholic liver disease, and most likely, nonalcoholic fatty liver disease [2]. Recently, it has been reported that higher level of oxidative stress and inflammation play a key role in the progression of HCC [3]. Oxidative stress may increase via the generation of reactive oxygen species (ROS) and defects in redox defense mechanisms with antioxidant enzymes, such as superoxide dismutase (SOD), catalase (CAT), or glutathione peroxidase (GPx), in mitochondria [4]. This mitochondrial dysfunction may lead to pathological mechanisms of chronic hepatic inflammation and subsequent hepatocarcinogenesis [3]. A case-control study has recently found that patients with HCC had significantly higher levels of oxidative stress, inflammation, and lower antioxidant capacities [5]. Consequently, it is worth trying an agent that can lower oxidative stress and reduce inflammation in patients with HCC.

Coenzyme Q10 (also called ubiquinone) is a lipid-soluble benzoquinone that has 10 isoprenyl units in its side chain and is a key component of the mitochondrial respiratory chain for adenosine triphosphate synthesis [6, 7]. Additionally, coenzyme Q10 is an intracellular antioxidant that protects membrane phospholipids, mitochondrial membrane protein, and LDL-C from free radical-induced oxidative damage [8, 9]. In vivo studies have demonstrated that coenzyme Q10 has therapeutic and chemotherapeutic effects by modulating the expression of hepPar-1, alpha-fetoprotein, inducible nitric oxide synthase, cyclooxygenase-2 and nuclear factor-κB (NF-κB) in the liver tissue of rats with HCC [10, 11]. However, to date, no published study has investigated the anti-oxidative and anti-inflammation effects of coenzyme Q10 supplementation in patients with HCC. Thus, the purpose of this clinical study was to examine the levels of oxidative stress, antioxidant enzymes activity, and inflammation after coenzyme Q10 supplementation (300 mg/day) in patients with HCC.

## Methods

### Participants

This study was designed as a single-blinded, randomized, parallel, placebo-controlled study. Following tumor resection, patients who were diagnosed with primary HCC (International Classification of Diseases 9, code 155.0) were recruited from the Division of General Surgery of Taichung Veterans General Hospital. We excluded patients who were younger than 20 years of age or older than 80 years of age, as well as during pregnant or lactating women, patient undergoing chemotherapy or hormone therapy, those with a history or current diagnosis of cardiovascular or renal disease, and those taking warfarin or statin medications. For patients taking vitamins supplements, we asked them to stop taking the supplements for at least for 1 month before enrolling in the study. Informed consent was obtained from each subject. This study was approved by the Institutional Review Board of Taichung Veterans General Hospital, Taiwan and registered at Clinical Trials.gov (NCT01964001).

Using a sample size calculation, we expected that the change in the levels of malondialdehyde would be 0.15 ± 0.2 μM after the coenzyme Q10 supplementation; therefore, the desired power was set at 0.8 to detect a true effect and at an α value equal to 0.05, with a minimum of 16 subjects in each intervention group. We enrolled 41 HCC patients in this study, and we used a random numbers table to randomly assign the subjects to the placebo (*n* = 20) or coenzyme Q10 (Q10-300 group, *n* = 21) group. The coenzyme Q10 and placebo (starch) capsules were commercially available preparations (New Health Co., Ltd. Taichung, Taiwan). The intervention was administered for 12 weeks. The subjects were instructed to take two capsules daily (coenzyme Q10 supplements 300 mg/d, 150 mg/b.i.d). To monitor compliance, we determined the degree of compliance for each patient, according to the number of returning capsules, and we measured the coenzyme Q10 level every 4 weeks after the supplementation. The following data were recorded for all subjects: age, blood pressures, and smoking and drinking habits, and exercise frequency. Body weight, height, waist and hip circumferences were measured; and the body mass index (BMI) and the ratio of waist to hip were calculated.

### Blood collection and biochemical measurements

Fasting venous blood samples (15 mL) were obtained to estimate each patient’s hematological and vitamin status. Blood specimens were collected in Vacutainer tubes (Becton Dickinson, Rutherford, NJ, USA) that contained EDTA as an anticoagulant or that contained no anticoagulant as required. Serum and plasma were prepared after centrifugation (3000 rpm, 4 °C, 15 min) and were then stored at -80 °C until analysis. Hematological entities, such as blood urea nitrogen, creatinine, glutamic oxaloacetic transaminase, glutamic pyruvate transaminase (GPT), and lipid profiles were measured using an automated biochemical analyzer (Hitachi-7180E, Tokyo, Japan). The level of high sensitivity C-reactive protein (hs-CRP) was quantified by particle-enhanced immunonephelometry with an image analyzer (Dade Behring, IL, USA). Plasma tumor necrosis factor-α (TNF-α) (R&D Systems Inc., Minneapolis, USA) and interleukin-6 (IL-6) (eBioscience, CA, USA) levels were measured using an enzyme-linked immunosorbent assay (ELISA) with commercially available kits, according to the manufacturer’s instructions.

Plasma coenzyme Q10 and vitamin E levels were measured using high-performance liquid chromatography (HPLC) and were detected with a UV detector at 275 nm and 292 nm, respectively [12, 13]. Plasma malondialdehyde (MDA) was determined using the TBARs (thiobarbituric acid reactive substances) method, as described by Botsoglou [14]. The red blood cell (RBC) samples were washed with normal saline after removing the plasma. Then, the RBC samples were diluted with 25x sodium phosphate buffer for superoxide dismutase (SOD) and glutathione peroxidase (GPx) measurements, with 250x sodium phosphate buffer for the catalase (CAT) measurement. The antioxidant enzymes activities (CAT, SOD, and GPx) were determined in the fresh samples and the methods used to measure these activities have been previously described [15–17]. The protein content of the plasma and RBC was determined based on the biuret reaction of the bicinchoninic acid (BCA) kit (Thermo, Rockford, IL, USA). The values of the antioxidant enzymes activities were expressed as unit/mg of protein. All analyses were performed in duplicate.

### Statistical analyses

The data were analyzed using SigmaPlot software (version 12.0, Systat, San Jose, CA, USA). The normality of the distribution of the variables was evaluated using the Shapiro-Wilk test. When comparing the continuous variables between the placebo and the Q10-300 groups, Student’s *t*-test or the Mann-Whitney rank sum test was used. For categorical response variables, the differences between the two groups were assessed using the Chi-square test or Fisher’s exact test. When comparing the levels of coenzyme Q10, vitamin E, and antioxidant enzymes activities at week 0, 4, 8, and week 12 within the group, one-way repeated measures ANOVA or Friedman repeated measures ANOVA on ranks was performed, and used Holm-Sidak post hoc test to assess the statistically significant differences. When comparing the levels of inflammatory markers at week 0 and week 12 within the group, paired-*t* test was performed. To examine the correlation between the coenzyme Q10 level and the levels of vitamin E, antioxidant enzymes activities, and inflammatory markers after the supplementation, Spearman rank order correlation was used. Additionally, simple linear regressions were used to examine the correlations between the changes in the levels of coenzyme Q10 and the changes in the levels of oxidative stress, antioxidant enzymes activity, and vitamin E. The results were considered to be statistically significant at *P* < 0.05. The values presented in the text are the means ± standard deviation (medians).

## Results

### Characteristics and dietary intake of subjects

**Fig. 1:**
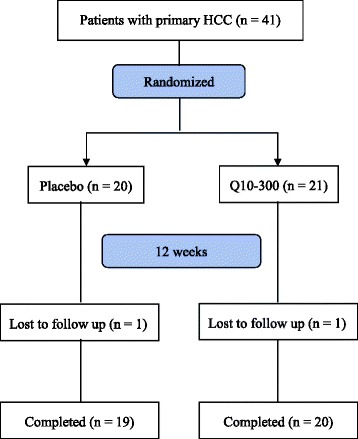
Flow Diagram

The sampling and trial profiles are summarized in Fig. 1, along with the number of subjects who completed the study in each group. Thirty-nine subjects completed the intervention (placebo, *n* = 19; Q10-300, *n* = 20). Table 1 shows the demographic characteristics and the dietary intakes of the subjects. At baseline, there were no significant between group differences in terms of age, blood pressure, anthropometric measurements, hematological entities, and dietary intake, as well as the frequency of smoking, drinking, or exercise. A total of 66.7 % of the subjects had hepatitis B, and 28.2 % of the subjects had hepatitis B or cirrhosis. In addition, 82.0 % of the subjects were newly diagnosed with HCC.

**Table 1:** Characteristics of subjects

	Placebo (*n* = 19)	Q10-300 (*n* = 20)
males (*n*, %)	12 (63.2 %)	15 (75.0 %)
age (y)	61.5 ± 11.0 (62.0)^a^	59.7 ± 8.3 (58.5)
SBP (mmHg)	133.8 ± 18.0 (134.0)	124.8 ± 19.3 (119.5)
DBP (mmHg)	77.9 ± 11.2 (78.0)	76.9 ± 12.3 (76.0)
waist circumference (cm)	89.1 ± 7.8 (88.0)	86.7 ± 10.2 (88.5)
waist hip ratio	0.92 ± 0.05 (0.91)	0.94 ± 0.16 (0.94)
BMI (kg/m^2^)	24.7 ± 3.2 (24.5)	23.1 ± 2.6 (22.8)
current smoker^b^, *n* (%)	6 (31.6 %)	5 (25.0 %)
drinks alcohol^c^, *n* (%)	3 (15.8 %)	3 (15.0 %)
Exercise^d^, *n* (%)	9 (47.4 %)	8 (40.0 %)
Hepatitis B, *n* (%)	14 (73.7 %)	12 (60.0 %)
Hepatitis C, *n* (%)	4 (21.1 %)	7 (35.0 %)
Cirrhosis, *n* (%)	6 (31.6 %)	5 (25.0 %)
Recurrence, *n* (%)	3 (15.8 %)	4 (20.0 %)
Hematology		
BUN (mg/dL)	15.5 ± 4.9 (15.0)	17.1 ± 9.2 (15.5)
creatinine (mg/dL)	0.8 ± 0.3 (0.8)	0.9 ± 0.3 (0.9)
GOT (IU/L)	35.2 ± 24.8 (27.0)	40.4 ± 23.0 (31.5)
GPT (IU/L)	44.8 ± 36.6 (38.0)	55.5 ± 39.8 (45.5)
TC (mg/dL)	162.7 ± 36.4 (148.0)	162.1 ± 32.7 (156.0)
TG (mg/dL)	95.3 ± 42.3 (87.0)	93.4 ± 29.9 (92.5)
LDL-C (mg/dL)	100.4 ± 32.3 (90.0)	102.3 ± 32.8 (100.0)
HDL-C (mg/dL)	50.6 ± 11.6 (50.0)	49.2 ± 12.4 (47.5)
TC / HDL-C	3.3 ± 1.0 (2.9)	3.5 ± 1.0 (3.5)
Dietary intake		
Energy (kcal/d)	1608.5 ± 334.5 (1531.2)	1686.5 ± 460.8 (1655.8)
Protein (g/d)	65.9 ± 17.9 (68.0)	66.2 ± 21.2 (66.8)
% of total calories	16.6 %	15.7 %
Fat (g/d)	49.5 ± 23.9 (48.2)	50.6 ± 16.1 (51.7)
% of total calories	26.6 %	26.8 %
carbohydrate (g/d)	228.6 ± 40.8 (227.0)	251.1 ± 89.6 (238.6)
% of total calories	57.2 %	57.9 %
PUFA (g/d)	15.6 ± 11.8 (13.0)	13.8 ± 8.6 (11.1)
MUFA (g/d)	15.1 ± 9.5 (11.9)	15.3 ± 6.6 (15.6)
SFA (g/d)	12.8 ± 8.0 (10.3)	14.2 ± 5.7 (14.3)
vitamin A (μg R.E./d)	3216.8 ± 4244.7 (1188.1)	3204.2 ± 3705.5 (1110.4)
vitamin E (mg α-T.E./d)	421.6 ± 727.1 (21.5)	615.0 ± 989.3 (10.8)
cholesterol (mg/d)	250.0 ± 163.8 (238.2)	231.8 ± 117.6 (197.8)
dietary fiber (g/d)	13.0 ± 6.5 (11.9)	10.9 ± 6.1 (9.7)

*BMI* body mass index, *BUN* blood urea nitrogen, *GOT* glutamic oxaloacetic transaminase, *HCC* hepatocellular carcinoma, *HDL*-*C* high-density lipoprotein-cholesterol, *LDL*-*C* low density lipoprotein-cholesterol, *MUFA* monounsaturated fatty acid, *PUFA* polyunsaturated fatty fat, *SFA* saturated fatty acid, *TC* total cholesterol, *TG* triglyceride

^a^means ± SD (medians)

^b^current smoker: individuals currently smoking one or more cigarettes per day

^c^drinks alcohol: individuals drinking one or 1 more alcoholic drinks per day regularly

^d^exercise: individuals who exercise regularly at least 3 times every week

### Levels of coenzyme Q10, antioxidant capacity, and inflammation after supplementation

**Fig. 2:**
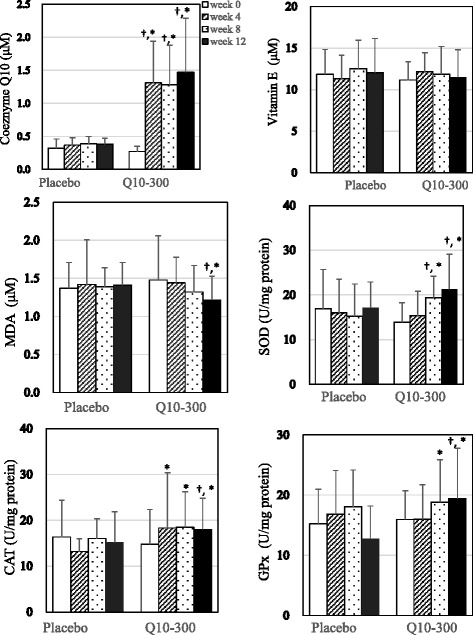
Levels of coenzyme Q10, vitamin E, oxidative stress, and antioxidant enzymes after supplementation. ^†^values are significantly different between the two groups. ^*^values are significantly different within each group. CAT, catalase activity; GPx, glutathione peroxidase; HCC, hepatocellular carcinoma; MDA, malondialdehyde; SOD, superoxide dismutase

The levels of coenzyme Q10, vitamin E, oxidative stress, and antioxidant enzymes after intervention are shown in Table 2 and Fig. 2. The plasma coenzyme Q10 concentration was significantly increased after the coenzyme Q10 supplementation (*P* < 0.01). Subjects in the Q10-300 group had significantly higher levels of coenzyme Q10 than those in the placebo group at weeks 4, 8, and 12 (*P* < 0.01). The plasma MDA level was significantly decreased after the coenzyme Q10 supplementation (1.48 ± 0.58 μM decreased to 1.22 ± 0.31 μM, *P* = 0.04) and in the patients in the placebo group at week 12 (1.22 ± 0.31 μM vs. 1.41 ± 0.30 μM, *P* = 0.04). In terms of the antioxidant enzymes activity, the SOD (13.93 ± 4.35 U/mg protein increased to 19.39 ± 4.79 U/mg protein, *P* < 0.01) and GPx activities (15.94 ± 4.77 U/mg protein increased to 18.82 ± 7.03 U/mg protein, *P* < 0.01) were significantly increased after 8 weeks of coenzyme Q10 supplementation and the CAT activity was significantly increased after 4 weeks of coenzyme Q10 supplementation (14.74 ± 7.63 U/mg protein increased to 18.32 ± 12.03 U/mg protein, *P* < 0.01).

**Table 2:** Changes in the levels of coenzyme Q10, vitamin E, oxidative stress, antioxidant enzymes activities, and inflammation in HCC patients after supplementation

	Placebo (*n* = 19)			Q10-300 (*n* = 20)		
	Week 0	Week 12	Week 12-0^b^	Week 0	Week 12	Week 12-0
coenzyme Q10 (μM)	0.32 ± 0.14 (0.33)^a^	0.38 ± 0.09 (0.37)	0.11 ± 0.15 (0.09)	0.27 ± 0.08 (0.27)	1.47 ± 0.82 (1.58)^e,d^	1.19 ± 0.84 (1.35)^d^
vitamin E (μM)	11.84 ± 2.98 (11.66)	12.04 ± 4.13 (11.47)	0.20 ± 2.79 (0.51)	11.16 ± 2.20 (12.10)	11.52 ± 3.28 (10.54)	0.43 ± 2.29 (0.54)
*Oxidative stress*						
MDA (μM)	1.37 ± 0.34 (1.42)	1.41 ± 0.30 (1.37)	0.08 ± 0.35 (0.11)	1.48 ± 0.58 (1.33)	1.22 ± 0.31 (1.10)^e,d^	−0.27 ± 0.68 (−0.33)^d^
Antioxidant enzymes activity						
SOD (U/mg protein)	16.94 ± 8.78 (13.10)	17.12 ± 5.79 (16.90)	0.73 ± 11.37 (3.00)	13.93 ± 4.35 (13.16)	21.26 ± 7.86 (22.02)^e,d^	7.96 ± 7.15 (7.50)^d^
CAT (U/mg protein)	16.36 ± 7.97 (12.00)	15.24 ± 6.62 (12.80)	−1.71 ± 6.25 (0.29)	14.74 ± 7.63 (11.75)	18.00 ± 6.82 (16.76)^e,d^	3.28 ± 7.76 (4.80)^d^
GPx (U/mg protein)	15.22 ± 5.76 (14.66)	12.73 ± 5.46 (12.20)	−1.10 ± 7.26 (−0.60)	15.94 ± 4.77 (17.46)	19.40 ± 8.34 (22.08)^e,d^	4.08 ± 7.67 (4.70)^d^
Inflammatory markers						
hs-CRP (mg/L)	3.5 ± 3.6 (1.6)	2.1 ± 3.9 (0.8)	−1.5 ± 4.2 (−0.8)	4.2 ± 4.2 (2.8)	2.6 ± 4.2 (1.0)^e^	−1.7 ± 4.8 (−1.3)
TNF-α (pg/mL)	0.89 ± 0.71 (1.16)	1.77 ± 4.38 (0.73)	0.88 ± 4.59 (−0.10)	0.84 ± 0.78 (0.73)	1.06 ± 1.02 (0.86)	0.30 ± 1.19 (0.02)
IL-6 (pg/mL)	3.76 ± 2.30 (3.44)	2.83 ± 3.64 (1.34)	−1.05 ± 3.72 (−1.65)	3.07 ± 2.03 (1.98)	1.62 ± 0.75 (1.70)^e^	−0.49 ± 2.96 (−0.70)

*CAT* Catalase activity, *GPx* glutathione peroxidase, *HCC* hepatocellular carcinoma, *hs*-*CRP* high sensitivity C-reactive protein, *IL*-*6* interleukin-6, *MDA* Malondialdehyde, *SOD* superoxide dismutase, *TNF*-α tumor necrosis factor-α

^a^means ± SD (medians)

^b^Difference between week 12 and week 0

^d^values are significantly different between the two groups

^e^values are significantly different within each group

The post-supplementation changes in the levels of coenzyme Q10, vitamin E, oxidative stress, antioxidant enzymes activities, and inflammation in HCC patients are also shown in Table 2. After 12 weeks of supplementation, subjects in the Q10-300 supplementation showed an increased level of changes in their level of coenzyme Q10 (*P* < 0.01) and antioxidant enzymes activity (SOD, *P* = 0.03; CAT, *P* = 0.01; GPx, *P* < 0.01) than those in the placebo group. The levels of MDA (*P* < 0.01) and inflammatory markers were significantly lower in the Q10-300 group compared to the placebo group after 12 weeks of supplementation (hs-CRP, 4.2 ± 4.2 mg/L decreased to 2.6 ± 4.2 mg/L, *P* < 0.01; IL-6, 3.07 ± 2.03 pg/mL decreased to 1.62 ± 0.75 pg/mL, *P* < 0.01). However, the levels of vitamin E (*P* = 0.81) and inflammatory markers (hs-CRP, *P* = 0.64, TNF-α, *P* = 0.68, IL-6, *P* = 0.80), as well as the difference between week 12 and week 0 of inflammatory markers (hs-CRP, *P* = 0.58, TNF-α, *P* = 0.29, IL-6, *P* = 0.13) were not significantly different between the placebo and Q10-300 groups after supplementation.

### Correlations between coenzyme Q10, antioxidant capacity, and inflammation after supplementation

The correlations between coenzyme Q10, vitamin E, oxidative stress, antioxidant enzymes activities, and inflammation after supplementation are shown in Table 3. The plasma coenzyme Q10 concentration was significantly correlated with the levels of vitamin E (*r* = 0.30, *P* = 0.01), MDA (*r* = -0.43, *P* = 0.01), and antioxidant enzymes activities (SOD, *r* = 0.31, *P* = 0.01; CAT, *r* = 0.24, *P* < 0.05; GPx, *r* = 0.25, *P* = 0.04) after 12 weeks of supplementation. However, there was no significant association between the levels of coenzyme Q10 and inflammatory markers after supplementation.

**Table 3:** Correlations between coenzyme Q10 level and vitamin E, oxidative stress, antioxidant enzymes activity and inflammation in HCC patients after supplementation

	Coenzyme Q10 level (μM) *r* ^a^ (*p* values)
vitamin E (μM)	0.30 (0.01)
Oxidative stress	
MDA (μM)	−0.43 (0.01)
Antioxidant enzymes activity	
SOD (U/mg protein)	0.31 (0.01)
CAT (U/mg protein)	0.24 (< 0.05)
GPx (U/mg protein)	0.25 (0.04)
Inflammatory markers	
hs-CRP (mg/L)	0.12 (0.49)
TNF-α (pg/mL)	0.01 (0.96)
IL-6 (pg/mL)	0.00 (0.99)

*CAT* Catalase activity, *MDA* Malondialdehyde, *GPx* glutathione peroxidase, *HCC* hepatocellular carcinoma, *hs*-*CRP* high sensitivity C-reactive protein, *IL*-*6* interleukin-6, *SOD* superoxide dismutase, *TNF*-α tumor necrosis factor-α

^a^correlation coefficient

**Fig. 3:**
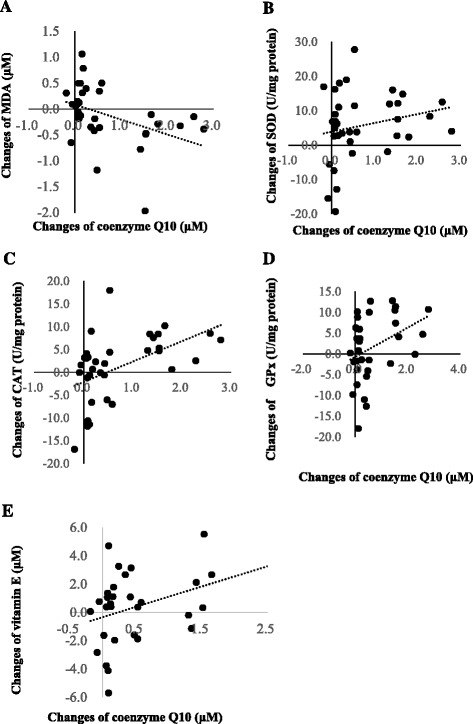
Correlations between changes in the levels of coenzyme Q10 and changes in the levels of oxidative stress, antioxidant enzymes activity, and vitamin E. **a** Correlation between the changes in the levels of coenzyme Q10 and MDA (*β* = -0.29, *p* = 0.02); **b** Correlation between the changes in the levels of coenzyme Q10 and SOD (*β* = 2.57, *p* = 0.02); **c** Correlation between the changes in the levels of coenzyme Q10 and CAT (*β* = 4.47, *p* < 0.01); **d** Correlation between the changes in the levels of coenzyme Q10 and GPx (*β* = 3.53, *p* = 0.03); **e** Correlation between the changes in the levels of coenzyme Q10 and vitamin E (*β* = 1.44, *p* = 0.01). CAT, catalase activity; GPT, glutamic oxaloacetic transaminase; GPx, glutathione peroxidase; HCC, hepatocellular carcinoma; MDA, malondialdehyde; SOD, superoxide dismutase

Furthermore, we examined the correlations between the changes in the levels of coenzyme Q10 and oxidative stress, antioxidant enzymes, and vitamin E (Fig. 3). Changes in the levels of coenzyme Q10 was significantly correlated with changes in the levels of MDA (*β* = -0.29, *p* = 0.02), antioxidant enzymes activity (SOD, *β* = 2.57, *p* = 0.02; CAT, *β* = 4.47, *p* < 0.01; *β* = 3.53, *p* = 0.03), and vitamin E (*β* = 1.44, *p* = 0.01).

## Discussion

This is the first clinical study to demonstrate that coenzyme Q10 (at a dose of 300 mg/d for 12 weeks) increases antioxidant enzymes activities (SOD, CAT, and GPx) and decreases oxidative stress and inflammation in patients with HCC. Coenzyme Q10 shows a significant anticarcinogenic activity in various cancer models via its antioxidant and anti-inflammatory properties [10, 11,18, 19]. Coenzyme Q10 is a highly safe and well tolerable supplement. Because coenzyme Q10 is a well antioxidant and anti-inflammation agent, it may represent a potential complementary therapeutic option in chronic disease patients who suffer from a higher status of oxidative stress or inflammation [20–22].

In this study, subjects in both groups had a low level of coenzyme Q10 (normal ranges: 0.5–1.7 μM) [23] at baseline (0.30 ± 0.11 μM). After a 4-week supplementation of coenzyme Q10 supplementation at a dose of 300 mg/d, the low level of coenzyme Q10 rapidly and significantly increased in patients with HCC (0.27 ± 0.08 μM to 1.31 ± 0.63 μM, *p* < 0.01), and this level was sustained for 12 weeks. Additionally, antioxidant enzyme activity significantly increased after the coenzyme Q10 supplementation, the CAT activity was significantly increased by 25.5 % after 4 weeks of supplementation, and SOD, CAT, and GPx were significantly increased by 43.2 %, 40.4 %, and 13.4 % after 8 weeks of supplementation. After 12 weeks of coenzyme Q10 supplementation, SOD, CAT, and GPx were significantly increased by 67.3 %, 42.6 %, and 26.5 % and oxidative stress (MDA) was significantly decreased by 17.3 %. Although there was no significant difference in the level of vitamin E after 12 weeks of supplementation, there was a significantly positive correlation between the levels of coenzyme Q10 and vitamin E after supplementation. Because patients with HCC suffer from a higher level of oxidative stress and inflammation [5], and a deficiency in coenzyme Q10 was found in HCC patients, we suggest that the administration of coenzyme Q10 supplements in patients with HCC during the surgery may be beneficial. Administered coenzyme Q10 at dose of 300 mg/d to HCC patients can significantly increase their antioxidant capacity, protect vitamin E against superoxide-driven oxidation and regenerate/maintain the level of vitamin E during antioxidation processes [24, 25].

Patients infected with hepatitis B and hepatitis C viruses account for 65–80 % of HCC worldwide [26]. In the present study, 66.7 and 28.2 % of the patients with HCC had a history of infection with hepatitis B and hepatitis C viruses, respectively. These chronic infections cause liver inflammation, and may lead to liver fibrosis/cirrhosis or HCC [27]. In this study, a total of 69.2 % of the subjects (*N* = 27, placebo, *n* = 11; Q10-300, *n* = 16) had chronic inflammation (hs-CRP ≥ 1.0 mg/L) and 38.5 % of the subjects (*N* = 15, placebo, *n* = 5; Q10-300, *n* = 10) had a higher level of inflammation (hs-CRP ≥ 3.0 mg/L) after surgery. After 12 weeks of coenzyme Q10 supplementation, the levels of hs-CRP and IL-6 were significantly decreased (Table 2), although the levels of inflammatory markers were not significantly lower compared to the placebo group and no significant association between the level of coenzyme Q10 and inflammatory markers after intervention (Table 3). We failure to detect significant correlations between the levels of coenzyme Q10 and inflammatory markers maybe due to higher variations of inflammation status in patients with HCC after surgery. Surgical resection is the most efficient treatment of patients with HCC. However, surgery may elicits the activation of the systemic inflammatory, endocrine/metabolic, and immunological systems, which is referred to as the surgical stress response [28]. However, Coenzyme Q10, as an antioxidant, could inhibit the inflammatory cascade of NF-κB -activation by the ROS [29–31]. Therefore, it may be useful to decrease inflammation in patients with HCC after surgery through coenzyme Q10 to increase the antioxidant capacity, particularly those with a higher level of inflammation.

Regarding the safety of coenzyme Q10 supplementation in the present study, there were no clinically significant changes in the subjects’ vital signs, serum chemical values, or hematological values, and there were no serious adverse events, no complaints of myalgia or muscle weakness, and no withdrawals due to adverse events. Note that the level of GPT was significantly decreased after the coenzyme Q10 supplementation (data not shown, 55.5 ± 39.8 IU/L decreased to 49.1 ± 46.2 IU/L, *p* = 0.02) and that changes in the levels of coenzyme Q10 were significantly correlated with changes in the levels of GPT (data not shown, *β* = -10.09, *p* = 0.03). In addition to GPT, the level of high density lipoprotein-cholesterol (HDL-C) was significantly increased after the coenzyme Q10 supplementation (data not shown, 1.3 ± 0.3 mmol/L increased to 1.5 ± 0.4 mmol/L, *p* < 0.01). Thus, at a dose of 300 mg/d coenzyme Q10 is safe for patients with HCC, and it protects against cardiotoxicity or liver toxicity during cancer treatment [32]. Our study has two limitations should be mentioned. First, the number of participants was small, although we did recruit more subjects than expected. Second, this study was designed for 12 weeks intervention only. Larger and longer intervention studies are needed to confirm the beneficial effect of coenzyme Q10 supplementation in patients with HCC after surgery.

## Conclusion

In conclusion, this clinical study is the first to demonstrate that a dose of 300 mg/d of coenzyme Q10 supplementation significantly increased antioxidant capacity and reduced the levels of inflammatory markers (hs-CRP and IL-6) in patients with HCC after surgery. We suggest that coenzyme Q10 supplementation could be considered as a complementary treatment strategy for patients with HCC after surgery, particularly those under higher levels of oxidative stress and inflammation.
